# Hierarchical Canonical Correlation Analysis Reveals Phenotype, Genotype, and Geoclimate Associations in Plants

**DOI:** 10.34133/2020/1969142

**Published:** 2020-03-31

**Authors:** Raphael Petegrosso, Tianci Song, Rui Kuang

**Affiliations:** Department of Computer Science and Engineering, University of Minnesota Twin Cities, Minneapolis, MN, USA

## Abstract

The local environment of the geographical origin of plants shaped their genetic variations through environmental adaptation. While the characteristics of the local environment correlate with the genotypes and other genomic features of the plants, they can also be indicative of genotype-phenotype associations providing additional information relevant to environmental dependence. In this study, we investigate how the geoclimatic features from the geographical origin of the *Arabidopsis thaliana* accessions can be integrated with genomic features for phenotype prediction and association analysis using advanced canonical correlation analysis (CCA). In particular, we propose a novel method called hierarchical canonical correlation analysis (HCCA) to combine mutations, gene expressions, and DNA methylations with geoclimatic features for informative coprojections of the features. HCCA uses a condition number of the cross-covariance between pairs of datasets to infer a hierarchical structure for applying CCA to combine the data. In the experiments on *Arabidopsis thaliana* data from 1001 Genomes and 1001 Epigenomes projects and climatic, atmospheric, and soil environmental variables combined by CLIMtools, HCCA provided a joint representation of the genomic data and geoclimate data for better prediction of the special flowering time at 10°C (FT10) of *Arabidopsis thaliana*. We also extended HCCA with information from a protein-protein interaction (PPI) network to guide the feature learning by imposing network modules onto the genomic features, which are shown to be useful for identifying genes with more coherent functions correlated with the geoclimatic features. The findings in this study suggest that environmental data comprise an important component in plant phenotype analysis. HCCA is a useful data integration technique for phenotype prediction, and a better understanding of the interactions between gene functions and environment as more useful functional information is introduced by coprojections of multiple genomic datasets.

## 1. Introduction

With the new high-throughput genomic array and sequencing technologies, large-scale genomic datasets in *Arabidopsis thaliana* have been greatly augmented or first become available in the last few years. The early large-scale genomic study in [1] used a 250 k SNP chip with multiple markers in each haplotype block to genotype a regional map (RegMap) of 1,307 accessions and conducted a global comparison of the traits in genetically distinct groups. Later, [2] extended this effort by applying whole-genome sequencing on 1,135 accessions to extract a map of more than 10 million biallelic SNPs and more than 1 million small-scale indels in the accessions. The large-scale genome resources from the studies enabled more comprehensive GWAS studies by the research community. More recent studies [3, 4] reported DNA methylomes of a global set of 144 accessions and a focused regional set of 150 Swedish accessions. In particular, the 1001 Epigenomes project [5] presented a larger comprehensive resource with 1,107 single-base resolution methylomes of 1,028 accessions and 1,203 transcriptomes of 998 accessions. Altogether, these resources have enabled more studies leading to better understandings of how the variations contribute to the molecular and nonmolecular phenotypes of *Arabidopsis thaliana*.

Large-scale efforts have also been made to catalog traits, phenotypes, and other characteristics of the species. Planteome [6] categorizes relations among traits (PTO), environmental conditions (PECO), and units of measurement (UO) in several ontologies. AraPheno [7] provides a repository of 288 phenotypes by the integration of the information and data in 17 studies, involving more than 7,000 accessions. The large amount of information in these resources has revealed important phenotype variabilities across the accessions. In a recent study in [8], a platform, CLIMtools, was developed to provide an integrative analysis of the correlations among genotypes, phenotypes, and geoclimate variables from the geographical origin of the accessions.

While the recent advances in [5, 8] have analyzed the genome and methylome variations for associations with phenotypes and geoclimate variables, these studies are limited to standard single-variable GWAS and correlation analysis. In this work, we integrate heterogeneous genomic data including transcriptome, methylome, and genotypes, together with the accessions' geoclimate background information to capture the interactions among the four types of variables for training models to predict phenotypes. To integrate the multiple genomic datasets and geoclimate dataset, we propose a novel method, namely, hierarchical canonical correlation analysis (HCCA), which integrates pairs of datasets hierarchically using the concept of condition number of the cross-covariance between a pair of datasets to determine the hierarchy. In this study, HCCA learns the joint feature representation between heterogeneous genomic data and geoclimate data to predict the special flowering time at 10°C (FT10) with supervised learning. In addition, we incorporate protein-protein interaction (PPI) Network with HCCA to find a coprojected feature representation that not only relies on the correlation among datasets but also retains the relationship among the proteins in the PPI to identify genes with consistent functions associated with the geoclimatic variables of interest.

The goal of this research is to study the role of geoclimatic features in plant genotype-phenotype association analysis. In particular, we investigated (1) whether the geoclimatic features are predictive of the flowering time phenotype and (2) whether the geoclimatic features are complimentary with the genomic features to improve phenotype predictions and (3) novel computational methods to integrate geoclimatic and genomic features to discover their associations and predict phenotypes. The contributions and the discoveries in this work are the following:

- We present a new approach (HCCA) to model the correlation structure among multiple datasets for an improved integrative analysis of genomic data with geoclimate data
- Our analysis confirms that geoclimatic features provide important information for the task of predicting the phenotype and improve the prediction by integration with genomic features
- The experiments tested several methods for coprojection of multiple datasets including hierarchical CCA, tensor CCA, and pairwise CCA with a comprehensive evaluation
- The experiments also show that enrichment analysis with the hierarchical coprojection of multiple genomic data analysis can identify more enriched GO terms associated with the geoclimatic features and, furthermore, a PPI network can be integrated in the coprojection by HCCA to improve the confidence of the enrichment analysis

The rest of the article is organized as follows: first, Section 2 describes experimental design, data preparation, the HCCA method to combine genomic data with geoclimate data, supervised learning for phenotype prediction, and the analysis of correlation in the coprojections. In Section 3, we show the experimental results of predicting the flowering time at 10°C using different combinations of the genomic datasets and the geoclimate dataset. This section also includes the enrichment analysis of the candidate genes closely associated with several geoclimatic features identified by HCCA. Finally, we discuss our work in Section 4.

## 2. Materials and Methods

**Figure 1:**
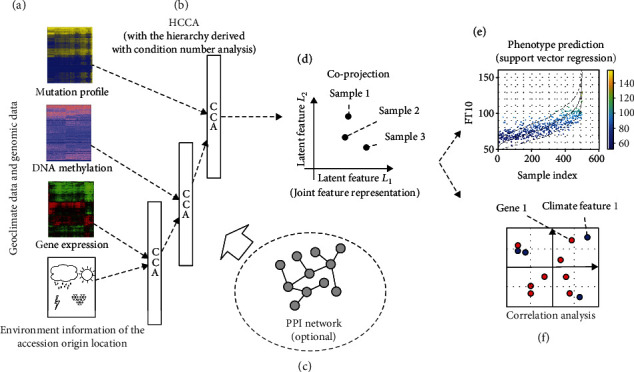
Overview of the workflow. (a) We first prepared the multiple genomic datasets and the geoclimate dataset. (b) HCCA is then applied to combine the information for finding a joint feature representation by coprojection of the datasets in a hierarchy learned by the analysis of the conditional numbers. (c) Optionally, the PPI network can also be integrated with HCCA to learn a more joint feature representation with better functional coherence. (d) The coprojection of the integrated data provides the joint feature representation of the integrated datasets. (e) The joint feature representation is then used by support vector regressor for phenotype prediction. (f) The joint feature representation is also analyzed for the association between genomic features and the geoclimatic features.

Figure 1 shows an overview of our workflow. Three genomic datasets of gene expressions, mutations, and DNA methylations are preprocessed and combined with matched accessions, and the geoclimatic variables of the origin of the accessions are collected in the geoclimate dataset shown in Figure 1(a), based on the assumption that coprojection of the multiple types of features will preserve true signals and remove the noise that might exist in each individual dataset. The joint representation obtained by the coprojection will contain more relevant information for predicting the phenotype. Figure 1(b) illustrates the structure of HCCA. HCCA applies CCA on each pair of datasets organized in a hierarchy of the four datasets to be coprojected. At each level of the hierarchy, the merge is decided by the analysis of condition numbers between each pair of the datasets. After applying the HCCA, a joint representation of the combined data is learned as shown in Figure 1(d). Note that HCCA also allows the option to incorporate the PPI network to impose functional coherence in the projection of the genomic features as shown in Figure 1(c). Next, the joint feature representation is used by a supervised learning algorithm such as support vector regression (SVR) to predict the continuous phenotype measure in Figure 1(e). In addition, the joint feature representation can also be used to analyze the canonical factor loadings [9–11] to identify the correlation between features, e.g., how well a gene correlates with a climate feature as shown in Figure 1(f).

### 2.1. Data Processing

The details of the datasets and data preprocessing used in the experiment are described below:

- *Accessions and phenotype annotations*. We downloaded from AraPheno [7] a dataset containing annotations of 7,425 accessions of *Arabidopsis thaliana*, including geographical location and phenotypes reported in 13 studies. Among the phenotypes, the flowering time at 10°C (FT10) was reported for 1,162 accessions and at 16°C (FT16) for 1,122 accessions by [5]. Since FT10 and FT16 are highly correlated (Pearson correlation = 0.88), our experiments in this study focus on the prediction and analysis of FT10 with additional results for validation on the FT16 phenotype shown in the Supplementary Figure 3
- *Gene expression data*. RNA-seq profiling of 727 accessions grown at 22° was downloaded from [5]. The transcriptomes were obtained with Illumina RNA sequencing, and report the RUVg normalized read counts processed by DESeq2 package [12] for variance stabilizing. We applied log10 transformation after adding a pseudocount of 1 to remove the skewness of the distribution. The accession Hi-0 (7167) was removed from the analysis since it is not included in AraPheno
- *Methylation data*. MethylC-seq data was downloaded from [5], containing 927 accessions, in which 777 intersect with AraPheno. We summarize the total number of reads at the methylated sites reported for each of the 28,496 genes in TAIR10 annotation including within the gene loci and also a neighborhood of 500 bps upstream and downstream. The counts were then transformed into *z*-scores across the samples to standardize the gene variance
- *Mutation data*. Mutation profiles were downloaded from the 1001 Genomes Consortium [2], containing more than 10 million biallelic SNPs of 1,135 accessions obtained from whole-genome sequencing. Since many genes are mutated in the profiles, we counted the number of mutations per gene using TAIR10 annotation to summarize the accumulated mutations of each gene, which is then normalized by *z*-score transformation across the samples to standardize the gene variance
- *Geoclimatic variables*. We downloaded geoclimatic variables of 1,131 accessions from CLIMtools [8]. CLIMtools compile 204 variables from a collection that includes climatic, atmospheric, and soil environmental variables from several sources. We removed 57 variables which contain missing entries in the accessions. Supplementary Table 1 shows the complete list of the used variables. It is important to note that the geoclimatic features do not represent the grown environment of the accessions but rather the environment of the location where accession originates
- *Protein-protein interaction network*. Protein-protein interaction (PPI) network for *Arabidopsis thaliana* was obtained from STRING [13] containing about 11 million known and predicted interactions for 25,490 proteins. The interactions include both direct (physical) and indirect (functional) associations

Combining all the datasets left 501 accessions that have genomic data and geoclimate information, as well as FT10 phenotype reported by AraPheno. In each genomic dataset, we kept the 5,000 genes with the highest variance before normalization across the samples in the dataset. Note that each type of the data was always collected in a single study, i.e., phenotypes and mutations are from 1001 Genomes Consortium [2], and gene expressions and methylations are from the study in [5]. Thus, there is no batch effect among the accessions in any kind of genomic features to correct. The PPI network was filtered to only include the 5,000 genes in each dataset, giving 280,673; 210,207; and 285,747 interactions for the gene expression dataset, the DNA methylation dataset, and the mutation dataset, respectively.

### 2.2. Hierarchical Canonical Correlation Analysis of Multiple Datasets

**Figure 2:**
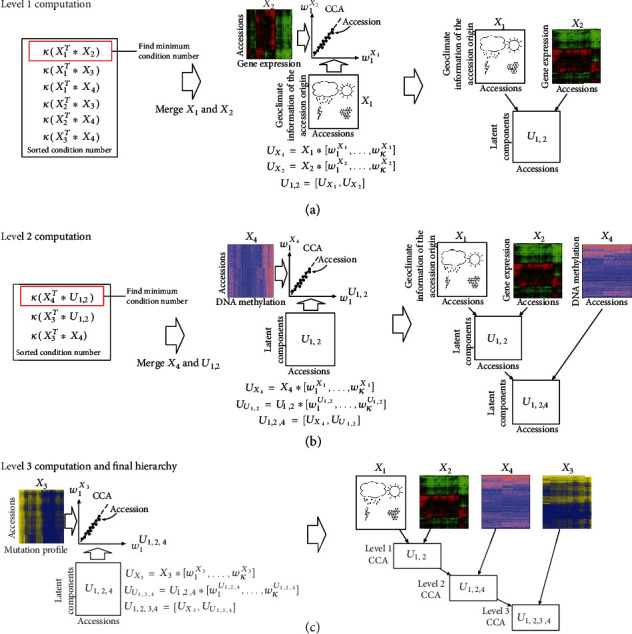
Hierarchical canonical correlation analysis. The figure shows the steps of HCCA. (a) At the first level, condition numbers are calculated for each pair of the four datasets. The pair (gene expression dataset *X*_1_ and geoclimate dataset *X*_2_) with the largest condition number is selected for performing CCA to find a feature representation that maximizes their correlation. (b) At level 2, condition numbers are calculated between DNA methylation dataset *X*_4_, mutation dataset *X*_3_, and the new dataset *U*_1,2_ combined from gene expression dataset *X*_1_ and geoclimate dataset *X*_2_ at level 1. The pair (DNA methylation dataset *X*_4_ and dataset *U*_1,2_) with the larger condition number is selected for coprojection into a new dataset *U*_1,2,4_ with CCA at this level. (c) At the last level, mutation data *X*_3_ and the dataset *U*_1,2,4_ are coprojected into the final combined dataset *U*_1,2,3,4_.

We propose a new method called hierarchical canonical correlation analysis (HCCA) to learn the joint feature representation for data integration.  Figure 2 shows the steps of running HCCA to coproject the four datasets: gene expression dataset *X*_2_, mutation dataset *X*_3_, DNA methylation dataset *X*_4_, and geoclimate dataset *X*_1_. HCCA applies canonical correlation analysis (CCA) to combine the datasets organized in a hierarchy derived from the datasets by calculating condition numbers. In this section, we first review CCA and explain the calculation of condition number and then introduce the HCCA algorithm.

#### 2.2.1. Canonical Correlation Analysis

Given two datasets, e.g., gene expression dataset *X*_2_ ∈ *ℝ*^*n*×*d*_2_^ and geoclimate dataset *X*_1_ ∈ *ℝ*^*n*×*d*_1_^, where *n* is the number of accessions and *d*_1_ and *d*_2_ are the number of features in the two datasets, respectively; the goal is to find a pair of projection vectors *w*_1_ ∈ *ℝ*^*d*1^ and *w*_2_ ∈ *ℝ*^*d*2^ such that the correlation between the canonical variables *u*_1_ = *X*_1_*w*_1_ and *u*_2_ = *X*_2_*w*_2_ is maximized in the following optimization problem:

(1) $$\begin{array}{c} \underset{w_{1},w_{2}}{\max}\text{corr}\left(u_{1},u_{2}\right)=\frac{u_{1}^{T}u_{2}}{\sqrt{u_{1}^{T}u_{1}u_{2}^{T}u_{2}}}=\frac{w_{1}^{T}C_{12}w_{2}}{\sqrt{w_{1}^{T}C_{11}w_{1}w_{2}^{T}C_{22}w_{2}}}, \end{array}$$

where *C*_*ij*_ = *X*_*i*_^*T*^*X*_*j*_. To obtain the optimal *w*_1_ and *w*_2_, it can be shown that CCA is equivalent to a generalized eigenvalue problem [14]. After obtaining the first pair of projection components *w*_1_ and *w*_2_, we can continue to find a second pair of projection components *w*_1_′ and *w*_2_′ by solving the same optimization problem with the additional constraints that *w*_1_′ and *w*_2_′ are orthogonal to *w*_1_ and *w*_2_, respectively. And, this process can be repeated to find a desired number of components for coprojection of the two datasets. The number of components to be chosen can be selected similarly as principal component analysis (PCA), in which the cumulative sum of the eigenvalues is smaller than a percentage on the total sum. For example, in our experiments, we set the percentage to be 85%. To remove the singularities in *C*_*ii*_ in the high-dimensional data, a *L*_2_ penalty on *w*_*i*_ can be introduced in the form of ${\tilde{C}}_{ii}=C_{ii}+\alpha I$ where parameter *α* > 0, similar to ridge regression [15–17] such that ${\tilde{C}}_{ii}$ becomes positive definite.

#### 2.2.2. Cross-Covariance Analysis with the Condition Number

As shown in Figure 2(a), HCCA first applies CCA between *X*_1_ and *X*_2_ to obtain a new joint representation *U*_1,2_ by concatenating the coprojection of *X*_1_ and *X*_2_ at level 1. After that, *U*_1,2_ and *X*_4_ are merged by a subsequent CCA to obtain a new representation *U*_1,2,4_ at level 2 shown in Figure 2(b). At the last level, a final CCA is performed between *X*_3_ and *U*_1,2,4_ to obtain the full joint representation *U*_1,2,3,4_ as shown in Figure 2(c). The key component of HCCA is to determine which pair of datasets to be coprojected with CCA at each level. We adopt the concept of condition number for the critical decision. Given matrix *A*, the condition number of *A*, *κ*(*A*), is defined as the following:

(2) $$\begin{array}{c} \kappa \left(A\right)=\left\|A\right\|*\left\|A^{-1}\right\|. \end{array}$$

If ‖·‖ is *L*_2_ norm and *A* is positive semidefinite, *κ*(*A*) can be rewritten as

(3) $$\begin{array}{c} \kappa \left(A\right)=\frac{\lambda_{\max}\left(A\right)}{\lambda_{\min}\left(A\right)}, \end{array}$$

where *λ*_max_(*A*) and *λ*_min_(*A*) are, respectively, the largest and smallest eigenvalues of *A*. The condition number of *A* measures how ill-conditioned *A* is. Accordingly, given *R* datasets *X*_1_ ∈ *ℝ*^*n*×*d*_1_^, ⋯, *X*_*R*_ ∈ *ℝ*^*n*×*d*_*R*_^, datasets *X*_*i*_ and *X*_*j*_ are chosen for coprojection if

(4) $$\begin{array}{c} \underset{i,j}{\arg\min}\kappa \left(X_{i}^{T}X_{j}\right),\text{such that }i,j\in \left\{1,\cdots ,R\right\}. \end{array}$$

If the condition number of *X*_*i*_^*T*^*X*_*j*_, *κ*(*X*_*i*_^*T*^*X*_*j*_) yields the minimum among all the pairs of the datasets, the cross-covariance matrix between *X*_*i*_ and *X*_*j*_ is the least similar to be singular.

#### 2.2.3. HCCA Algorithm

HCCA applies the heuristic to identify the strongest correlation among the datasets for reinforcing the signals from each other dataset. The process is repeated until all the datasets are integrated.  Algorithm 1 shows the steps for the computation of HCCA on *N* datasets.

In the algorithm, lines 5-13 find the indices of the datasets *X*_*iarg*_ and *X*_*jarg*_ with the smallest condition number of their cross-covariance for the next CCA. Line 14 calls the CCA procedure to find the joint representation *U*_*i*,*j*_ of the selected datasets, the datasets *X*_*iarg*_ and *X*_*jarg*_. Lines 15-17 replace *X*_*iarg*_ and *X*_*jarg*_ by their joint representation *U*_*i*,*j*_ in the array of the datasets. Finally, the HCCA function is called recursively on line 18 and the program will terminate when there is only one dataset left.

Let *d* be the largest feature dimension of all the datasets, finding the indices *i* and *j* has time complexity *𝒪*(*N*^2^*d*^3^) to find the condition number of all pairs of *N* datasets, and time complexity *𝒪*(*d*^3^) is needed to solve the generalized eigenvalue problem by singular value decomposition (SVD) for CCA. Therefore, given the number of datasets *N* ≪ *d*, the HCCA algorithm has the same asymptotic time complexity as the original CCA.

#### 2.2.4. Incorporating Protein-Protein Interaction Network

HCCA relies only on the correlation among the datasets to find a joint representation. This CCA-based approach, however, does not take into account the underlying relationship among the features (genes) in each genomic dataset, while known relations between the genes might guide the selection of the sets of genes that are more coherent in functions and less likely to include correlation between noisy signals. A natural source of information of gene relationship is protein-protein interactions in PPI networks. In our experiments, we utilized STRING [13] PPI network. Given graph *G*_*i*_ of a PPI network, its adjacency matrix *A*_*i*_ ∈ *ℝ*^*d*_*i*_×*d*_*i*_^ is defined on the features of *X*_*i*_, and the normalized graph Laplacian of *A*_*i*_ is defined as *L*_*i*_ = *I* − *D*_*i*_^−1/2^*A*_*i*_*D*_*i*_^−1/2^, where *D*_*i*_ is the degree matrix of *A*_*i*_. *L*_*i*_ can be utilized as a smoothness term on *w*_*i*_ using the following fact:

(5) $$\begin{array}{c} w_{i}^{T}L_{i}w_{i}=\frac{1}{2}\overset{d_{i}}{\underset{j,j^{\prime }=1}{\sum }}A_{jj^{\prime }}{\left(\frac{w_{i,j}}{\sqrt{D_{jj}}}-\frac{w_{i,j^{\prime }}}{\sqrt{D_{j^{\prime }j^{\prime }}}}\right)}^{2}. \end{array}$$

Note that *w*_*i*_^*T*^*L*_*i*_*w*_*i*_ enforces *w*_*i*,*j*_ and *w*_*i*,*j*′_ to have a similar value if *j* and *j*′ nodes have a strong connection in graph *G*_*i*_. The advantage of utilizing graph Laplacians of PPI networks for network-based feature selection has been well explored previously [18, 19]. We also propose, therefore, the use of network-based feature smoothing on CCA using the graph Laplacian of the PPI network as follows:

(6) $$\begin{array}{c} \underset{w_{i},w_{j}}{\max}\text{corr}\left(u_{i},u_{j}\right)=\frac{w_{i}^{T}C_{ij}w_{j}}{\sqrt{w_{i}^{T}\left(C_{ii}+\alpha_{i}I\right)w_{i}+\alpha_{i}w_{i}^{T}L_{i}w_{i}}\sqrt{w_{j}^{T}\left(C_{jj}+\alpha_{j}I\right)w_{j}+\alpha_{j}w_{j}^{T}L_{j}w_{j}}}, \end{array}$$

which is equivalent to the original CCA by making the update to each *C*_*ii*_ as ${\tilde{C}}_{ii}=C_{ii}+\alpha_{i}\left(I+L_{i}\right)$.

#### 2.2.5. Hyperparameter Tuning

Note that learning embedding or projection of the data is unsupervised; therefore, the hyperparameters *α* (when running CCA without PPI network) or *α*_*i*_s (when running CCA with PPI network) cannot be chosen by cross-validation, different from supervised learning or transductive learning as in [15]. Thus, in the application of the HCCA algorithm, we propose to also estimate the hyperparameters from cross-covariance analysis by the condition number.

When HCCA runs CCA without PPI network as in equation (1) with ${\tilde{C}}_{ii}=C_{ii}+\alpha I$, to make ${\tilde{C}}_{ii}$ well-conditioned, we choose *α* such that ${\tilde{C}}_{ii}$ has a desirable condition number using the technique of reconditioning [20]. Specifically, given *c* as the desirable condition number of ${\tilde{C}}_{ii}$, *α* can be chosen as

(7) $$\begin{array}{c} \kappa \left({\tilde{C}}_{ii}\right)=\frac{\lambda_{\max}\left(C_{ii}\right)+\alpha }{\lambda_{\min}\left(C_{ii}\right)+\alpha }=c⟹\alpha =\frac{\lambda_{\max}\left(C_{ii}\right)-c\lambda_{\min}\left(C_{ii}\right)}{c-1}. \end{array}$$

Note that, even though *c* is also a parameter to be chosen, it is more interpretable than *α*. As a rule of thumb, a matrix with a condition number smaller than 10 is considered well-conditioned [21]. Our experiments suggest that using *c* = 4 yields good overall results (see that the comparison in Supplementary Table 3 suggests *c* = 4 generates better cross-variance structure for HCCA but plays little role in PCCA and TCCA).

When HCCA runs CCA with PPI network as in equation (6), the hyperparameters are *α*_*i*_s. Since the eigenvalues of the normalized Laplacian matrix are between 0 and 2, *λ*_max_(*I* + *L*) ≤ 1 + 2, and *λ*_min_(*I* + *L*) ≥ 1 + 0 by the Weyl's inequality (given three symmetric matrices *A*, *B*, and *C* such that *C* = *A* + *B*, we have that *λ*_max_(*C*) ≤ *λ*_max_(*A*) + *λ*_max_(*B*) and *λ*_min_(*C*) ≥ *λ*_min_(*A*) + *λ*_min_(*B*), where *λ*_max_ and *λ*_min_ are the largest and smallest eigenvalues of the respective matrix). Accordingly, $\lambda_{\max}\left({\tilde{C}}_{ii}\right)\leq \lambda_{\max}\left(C_{ii}\right)+3\alpha$ and $\lambda_{\min}\left({\tilde{C}}_{ii}\right)\geq \lambda_{\min}\left(C_{ii}\right)+\alpha$. Therefore, we can obtain $\kappa \left({\tilde{C}}_{ii}\right)\leq c$ by

(8) $$\begin{array}{c} \kappa \left({\tilde{C}}_{ii}\right)=\frac{\lambda_{\max}\left({\tilde{C}}_{ii}\right)}{\lambda_{\min}\left({\tilde{C}}_{ii}\right)}\leq \frac{\lambda_{\max}\left(C_{ii}\right)+3\alpha }{\lambda_{\min}\left(C_{ii}\right)+\alpha }=c⟹\alpha =\frac{\lambda_{\max}\left(C_{ii}\right)-c\lambda_{\min}\left(C_{ii}\right)}{c-3}. \end{array}$$

### 2.3. Plant Phenotype Prediction

As shown in Figure 1(e), we predict the flowering time at 10°C (FT10) using the support vector regression (SVR) algorithm [22, 23]. Let $\tilde{X}\in {\mathbb{R}}^{n\times k}$ be the final combined dataset *U*_1,2,3,4_ learned by HCCA combining genomic datasets and geoclimate dataset. In this regression problem, we are interested in learning a regression function over the samples in $\tilde{X}$ to predict the FT10 of these samples as a vector *y* ∈ *ℝ*^*n*^, i.e., $f:\tilde{X}\left[i\right]⟶y_{i}$ for *i* = 1, 2, ⋯, *n*. SVR is a margin-insensitive regression algorithm allowing the use of kernel functions for nonlinear mapping. In our experiments, we used the Gaussian kernel *K*(*x*_*i*_, *x*_*j*_) = exp(−*σ*‖*x*_*i*_ − *x*_*j*_‖^2^).

### 2.4. Detecting Gene-Geoclimate Interactions by Canonical Factor Loadings

**Figure 3:**
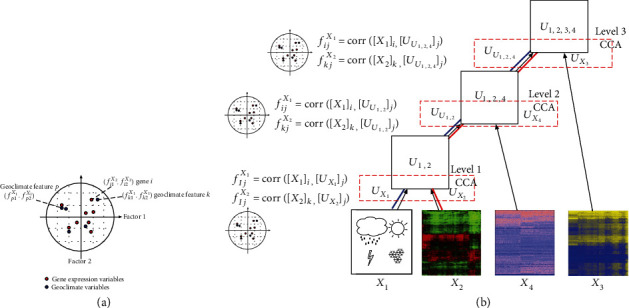
Analysis of gene-geoclimate interactions by canonical factor loadings. (a) The plot shows the projection of the factor loadings of genes and climate features on the first two canonical variables. In this example, gene *i* and geoclimatic feature *k* bear similar influence on the two canonical components. (b) Geoclimatic features and gene features can be associated by correlations with canonical factor loadings of the datasets at different levels of the hierarchy.

*Arabidopsis thaliana* is a suitable species for studying gene and geoclimate interactions [5, 8] because the species self-pollinates and has not been subject to selective breeding. We use canonical factor loadings (also called structure correlation coefficients) [9–11] to measure the importance of each original variable for the canonical variables found by CCA. Given input variables *X*_1_ and *X*_2_, e.g., geoclimatic variables and gene expressions, respectively, and the resulting canonical variables *U*_*X*_1__ and *U*_*X*_2__ learned by CCA, the canonical factor loading *f*_*ij*_ between a *i*th gene [*X*_2_]_*i*_ ∈ *ℝ*^*n*^ and the *j*th canonical variable [*U*_*X*_2__]_*j*_ ∈ *ℝ*^*n*^ is given by *f*_*ij*_^*X*_2_^ = corr([*X*_2_]_*i*_, [*U*_*X*_2__]_*j*_), where [*X*_2_]_*i*_ and [*U*_*X*_2__]_*j*_ are vectors in the sample space, and therefore, *f*_*ij*_^*X*_2_^ measures the correlation between the *i*th input variable in the original space and the *j*th canonical variable. Similarly, given a geoclimatic feature [*X*_1_]_*k*_, with respect to the same canonical variable [*U*_*X*_1__]_*j*_, the canonical factor loading *f*_*kj*_^*X*_1_^ = corr([*X*_1_]_*k*_, [*U*_*X*_1__]_*j*_).  Figure 3 visualizes the canonical factor loadings of gene expression variables and geoclimatic variables calculated with respect to the first 2 canonical factors. Assuming a transitive relation on correlations among random variables, *f*_*kj*_^*X*_1_^ and *f*_*ij*_^*X*_2_^ are close in the canonical factor loading space in Figure 3, and thus, geoclimatic variable [*X*_1_]_*k*_ and gene expression variable [*X*_2_]_*i*_ should also be highly correlated. In the example, geoclimatic feature *p* has a negative influence on the canonical component 1 and, therefore, does not correlate with gene *i*. In our experiments, we used the Euclidean distance between pairs of canonical factor loadings to find the associations between the variables.

In the case where HCCA is applied to coproject more than two datasets, the canonical factor loadings can also be measured at different levels of the hierarchical structure.  Figure 3(b) gives an example of when we are analyzing the correlation between gene expression and geoclimatic variables. In the figure, we can see that the red path shows how the information of the gene expression matrix *X*_2_ flows to the root of the hierarchy and, in blue, the information of geoclimatic variables flowing to the root. Therefore, the canonical factor loading *f*_*ij*_^*X*_2_^ of *X*_2_, with respect to component *j*, can be calculated as *f*_*ij*_^*X*_2_^ = corr([*X*_2_]_*i*_, *Z*), where *Z* ∈ {[*U*_*X*_2__]_*j*_, [*U*_*U*_1,2__]_*j*_, [*U*_*U*_1,2,4__]_*j*_}, which are the canonical variables in the path to the root. Similarly, *f*_*kj*_^*X*_1_^ can be found as *f*_*kj*_^*X*_1_^ = corr([*X*_1_]_*k*_, *W*), where *W* ∈ {[*U*_*X*_1__]_*j*_, [*U*_*U*_1,2__]_*j*_, [*U*_*U*_1,2,4__]_*j*_}. The possible equations, at each level, are also depicted at Figure 3(b).

### 2.5. Comparison with Pairwise CCA and Tensor CCA

There also exist two other CCA-based methods for learning joint projection of more than two datasets. One of the most common approaches is the maximization of the pairwise correlation among the datasets with pairwise CCA [24]:

(9) $$\begin{array}{c} \begin{array}{cc} \underset{w_{1},\cdots ,w_{N}}{\max} & \overset{N}{\underset{i,i^{\prime }=1;i>i^{\prime }}{\sum }}\frac{w_{i}^{T}C_{ii^{\prime }}w_{i^{\prime }}}{\sqrt{w_{i}^{T}C_{ii}w_{1}w_{i^{\prime }}^{T}C_{i^{\prime }i^{\prime }}w_{i^{\prime }}}},\\ \text{subject to} & w_{i}^{T}C_{ii}w_{i}=1,i=1,\cdots ,N. \end{array} \end{array}$$

More recently, [15] introduced a tensor CCA (TCCA) formulation which maximizes the high-order correlation among the datasets:

(10) $$\begin{array}{c} \begin{array}{cc} \underset{w_{1},\cdots ,w_{N}}{\max} & C_{12\cdots N}{\bar{\times }}_{1}w_{1}{\bar{\times }}_{2}w_{2}\cdots {\bar{\times }}_{N}w_{N},\\ \text{subject o} & w_{i}^{T}C_{ii}w_{i}=1,i=1,\cdots ,N, \end{array} \end{array}$$

where *𝒞*_12⋯*N*_ is the covariance tensor. It is shown that the problem can be solved using alternating least squares for CANDECOMP/PARAFAC (CP) decomposition of the tensor [15]. In spite of the success of pairwise CCA and TCCA in combining multiple datasets in other applications, they might not be particularly suitable for integrating high-dimensional genomic datasets. First, enforcing all the datasets to correlate simultaneously can be too restrictive if one or a subset of the datasets has a relatively low correlation with the other datasets. Second, there might be a group structure among the datasets, i.e., datasets inside the same group correlate very well while datasets in different groups correlate less. HCCA is specifically designed to introduce a hierarchy among the dataset to capture the structure and minimize information loss by coprojecting similar datasets first. In our experiments, we will demonstrate that HCCA outperforms pairwise CCA and TCCA by learning a more informative coprojection of gene expressions, DNA methylations, and mutations for FT10 phenotype prediction.

## 3. Results

In this section, we describe the experiments and the results of phenotype prediction and the correlation analysis among genomic and geoclimatic variables.

### 3.1. Prediction of Flowering Time

In this experiment, we compare HCCA with several other baseline methods to predict the flowering time at 10°C (FT10) phenotype collected from AraPheno. We first show the importance of including geoclimatic features from the accessions' origin location using CCA, and then, we present the improved prediction results using HCCA to integrate multiple datasets.

#### 3.1.1. Baselines

HCCA was compared with the following baselines in the task of predicting FT10 phenotype:

- Similarity network fusion (SNF) [25] combines multiple genomic data by building a similarity network for each of the data types and then integrating these networks using network fusion. Afterward, graph embedding by SVD is applied to the fused network to obtain the top *r* first components for feature embedding
- Pairwise CCA (PCCA), described in Section 2.5, maximizes the pairwise correlation across all the datasets simultaneously
- Tensor CCA (TCCA), described in Section 2.5, maximizes high-order tensor correlation across all the datasets
- Stacked datasets (Stacked). We also considered a naive approach that stacks all the datasets together in a single matrix. Specifically, the 4 datasets *X*_1_ ∈ *ℝ*^*n*×*d*_1_^, ⋯, *X*_4_ ∈ *ℝ*^*n*×*d*_4_^ are stacked to be a *U*_1,2,3,4_ ∈ *ℝ*^*n*×(*d*_1_ + *d*_2_ + *d*_3_ + *d*_4_)^.

For HCCA, PCCA, and TCCA, automatic tuning of parameters was performed using the mechanism described in Section 2.2.5. To run SNF, we performed a grid search on its parameters *K* = [10, 20, 30], *α* = [0.3, 0.4, ⋯, 0.9], and *T* = [10, 20] and the number of components in the embedding *r* = [10, 20, ⋯, 100] and report the best result obtained in the grid search.

#### 3.1.2. Evaluation

After obtaining the joint representation of all the datasets, the data was randomly partitioned into a training set and a test set. Each training set was used to train an SVR model using MATLAB function *fitrsvm* with Gaussian kernel. 10-fold cross-validation was performed on the training set for the selection of SVR parameters. After training the regression model with the training set, the test set was used to generate the coefficient of determination *R*^2^, which measures the goodness-of-fit of a regression model by the proportion of variance in the target variable *y* that can be explained by the model variables. *R*^2^ is defined by the following expression:

(11) $$\begin{array}{c} R^{2}=1-\frac{\sum_{i=1}^{n}{\left(y_{i}-{\overset{\wedge }{y}}_{i}\right)}^{2}}{\sum_{i=1}^{n}{\left(y_{i}-\overset{\wedge }{y}\right)}^{2}}, \end{array}$$

where *y*_*i*_s are the ground-truth values, ${\overset{\wedge }{y}}_{i}$ are the predicted values, and $\bar{y}$ is the mean of *y*_*i*_s on the training set. We repeat the experiments 200 times and report the mean and standard deviation of *R*^2^.

#### 3.1.3. Incorporation of Geoclimatic Data Improves Phenotype Prediction

We first analyzed the effect of combining geoclimatic variables of the accession locations with each type of genomic data to predict FT10 values. The assumption is that the FT10 phenotype is not only defined by the genomic features but also highly related to the environment of the original location which shaped the genetic makeup of the accession by evolution.

**Figure 4:**
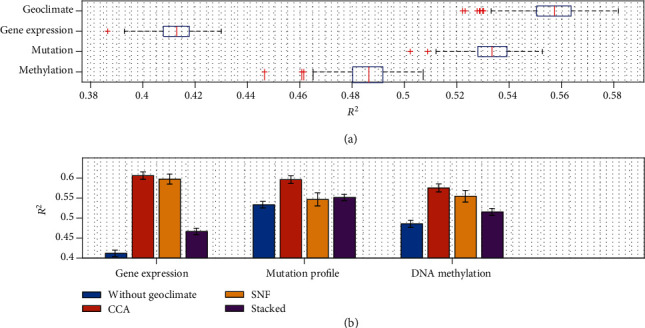
Improved FT10 phenotype prediction by the integration of geoclimate data. (a) Phenotype prediction accurate by learning with each individual genomic dataset or geoclimate dataset. (b) Phenotype prediction accurate by integrating one type of genomic data with geoclimate data.

Figure 4(a) shows the results by training with each individual dataset. Interestingly, training with the geoclimate dataset obtains the best mean *R*^2^ value 0.557, followed by the mutation dataset with a mean of 0.533. The result suggests that the flowering time is more predictable by the genotypes which are shared by the environment at the location of accession origin. Without a surprise, the gene expression dataset provides the least information for FT10 prediction with a mean *R*^2^ of 0.412 since both the gene expressions are collected from the samples grown in a different controlled environment from the samples used for FT10 phenotyping. Note that training using DNA methylation dataset results in a higher mean *R*^2^ value of 0.486 because DNA methylation is probably less affected by the different growth environment than gene expressions [5].

Figure 4(b) shows the results of combining each individual genomic dataset with the geoclimate dataset. Combining mutation profiles with geoclimatic variables by CCA resulted in a mean *R*^2^ value of 0.596, against 0.533 of using mutation profiles alone, and similar improvements are also observed in the combination with gene expressions and DNA methylations. CCA performs better than SNF and simple stacking of the datasets in every case. Clearly, stacking the datasets together does not capture the relationship among the datasets for improving the prediction. We also tested stacking the projected datasets with PCA. The results are worse (not shown). SNF seems to obtain better results in combining the genomic datasets such as mutation profile combined with gene expression data rather than the geoclimate dataset. The detailed results are shown in Supplementary Figure 1.

#### 3.1.4. Integrating All Genomic Datasets and Geoclimate Dataset Provides the Best Prediction

**Figure 5:**
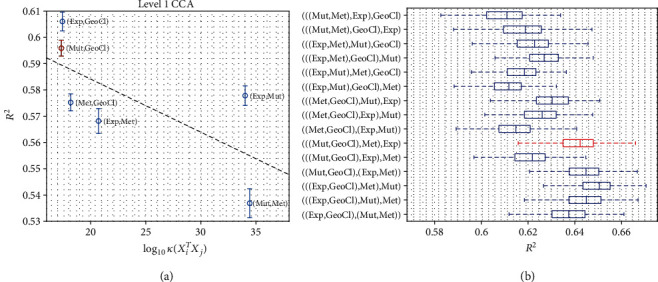
Learning hierarchical organization of the datasets by HCCA. (a) The *R*^2^ values of FT10 prediction by combinations of each pair of datasets are plotted against the condition number of the cross-covariance matrix of the two datasets. The *x*-axis shows the log of the condition number *κ*(*X*_*i*_^*T*^*X*_*j*_) of a pair of datasets (*X*_*i*_, *X*_*j*_), and the *y*-axis is the *R*^2^ of the prediction obtained by SVR. (b) Comparison of the different hierarchical organizations of the datasets used for integration by CCA. The red combination is selected by the analysis of condition numbers in HCCA.

We next show how multiple genomic datasets can be further integrated together with the geoclimate dataset to improve the prediction of FT10. To understand the role of the condition numbers used by HCCA to build the hierarchical organization of the datasets, we first analyzed how the relation between the prediction accuracy and the condition number *κ*(*X*_*i*_^*T*^*X*_*j*_) between each pair of the datasets *X*_*i*_ and *X*_*j*_ in Figure 5(a). The points in the figure represent all the pairs of the four datasets to be combined in the first level, and the dashed line is a linear fitting to the points. The negative trend (correlation −0.6934) is clear between the log of *κ* and the *R*^2^. HCCA selects the mutation dataset and the geoclimate dataset (shown in red) to be coprojected at level 1, which achieves the second best *R*^2^, only slightly after the best option (combining the gene expression dataset and the geoclimate dataset). The plot clearly suggests that the analysis of condition number is a useful strategy. Figure 5(b) compares different hierarchies to organize the datasets in HCCA. First, it appears that the prediction performance is sensitive to the order how the datasets are selected for coprojection at different levels since the mean *R*^2^ obtained is significantly different (ANOVA *p* value = 0). Second, marked in red, HCCA selects the fourth best order of combining the datasets while the two best options start with the selection of gene expressions and geoclimatic variables, both of which also generates a *κ* value very similar to the one selected by HCCA (mutation profile and geoclimatic variables) as shown in Figure 5(a), and the third best option also starts with the selection of mutation profile and geoclimatic variables.

**Figure 6:**
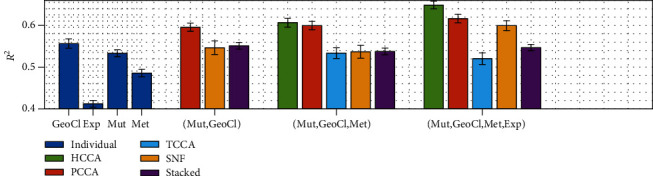
Improvement by integration of all the datasets. The figure shows the mean and variance of *R*^2^ reported for each combination of the datasets and data integration methods. The results of using the individual dataset are shown for comparison in the blue bar plots on the left. The *x*-axis shows the datasets used to generate the results in the bar plot, and the *y*-axis the mean and variance of *R*^2^ values obtained by SVR.

Figure 6 shows the results when multiple genomic datasets are integrated with HCCA and the baseline methods. In the plots, the results are shown for one to four datasets used for prediction from left to right. Overall, we observe the same pattern in all the compared methods that the more datasets integrated, the better the prediction is. HCCA, applied to integrate three datasets and four datasets, exhibits a better *R*^2^ value than the baseline methods, suggesting that the hierarchical integration of the datasets collaborates to extract more relevant signals than simultaneous integration of the datasets together in one step. It is interesting that the most restrictive project method, tensor CCA (TCCA), does not perform well as the other methods. We believe that TCCA fails to discover optimal correlation among the datasets since higher-order Pearson's correlation is not well-defined when inconsistent correlations exist among different subsets of the random variables. Stacking the datasets also does not perform very well even if the results are consistently improved as more datasets are considered. Finally, SNF performed relatively well but adding more datasets does not seem to play a large impact on the results.

**Figure 7:**
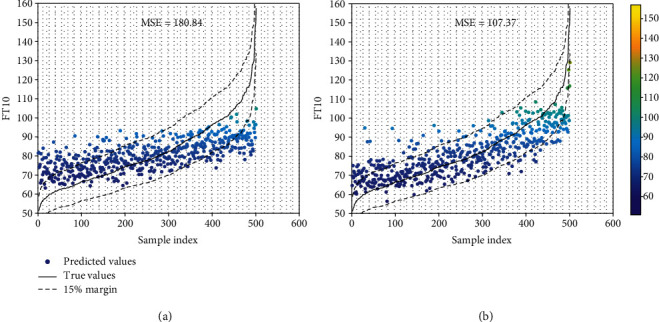
Visualizing predictions in comparison to the range of true values. The plots show how well the predicted values fit the ground-truth values in two cases: (a) using only gene expression data and (b) using all the four datasets by HCCA. In the plots, the *x*-axis represents the samples sorted by the true FT10 values, and *y*-axis the FT10 values. The ground-truth values are shown as the solid line, dashed lines represent a margin of 15%, and the dots represent the prediction of each sample. At the top, we also show the mean squared error (MSE) of the predictions.

Figure 7 shows a visual comparison of how well the predictions fit the ground-truth values. In the two cases, we can notice that SVR trained with gene expression only fails to predict the FT10 values in the 15% confidence range with an MSE of 180.84 while SVR trained on all the datasets integrated with HCCA predicts FT values well within the confidence range with an MSE of 107.37.  Table 1 also shows the proportion of the predictions that fall within 5%, 10%, and 15% confidence range, for each individual dataset, and the integrated four datasets by HCCA, PCCA, and TCCA. The results further confirm that integrating multiple datasets with HCCA significantly improves the predictions of FT10 values.

In Supplementary Figure 3, we also show prediction results of the FT16 values using the same experimental setting. Very similar results are observed in all the experiments with no surprise since the FT16 phenotypes are highly correlated with the FT10 phenotypes.

### 3.2. Detecting Gene-Geoclimate Associations

In this experiment, we evaluate how well HCCA can identify genes correlated with geoclimatic variables of interest. We also show that by incorporating the PPI network, we can further improve the relevance of the identified genes by leveraging the information of the interactions between their protein products. To measure the relevance of the associated genes, we performed gene enrichment with clusterProfiler [26], with a *p* value cutoff of 0.05 after Bonferroni correction. Two types of enrichment terms were considered: Biological Processes Gene Ontology (GO) terms [27, 28] and Kyoto Encyclopedia of Genes and Genomes (KEGG) pathway terms [29].

#### 3.2.1. Hierarchical Enrichment Analysis Detects More Relevant Gene-Geoclimate Associations

To detect gene expressions correlating with geoclimatic variables of interest, we fixed the first level of the hierarchical structure as coprojecting gene expressions and geoclimatic features such that their associations can be evaluated at every level in the hierarchy. Based on the condition numbers, DNA methylation is chosen to be merged at level 2 and then mutation profiles at level 3. After the coprojections, we calculated the canonical factor loadings for the geoclimatic features and gene expressions to project them together for analysis of correlation based on their proximity.

**Figure 8:**
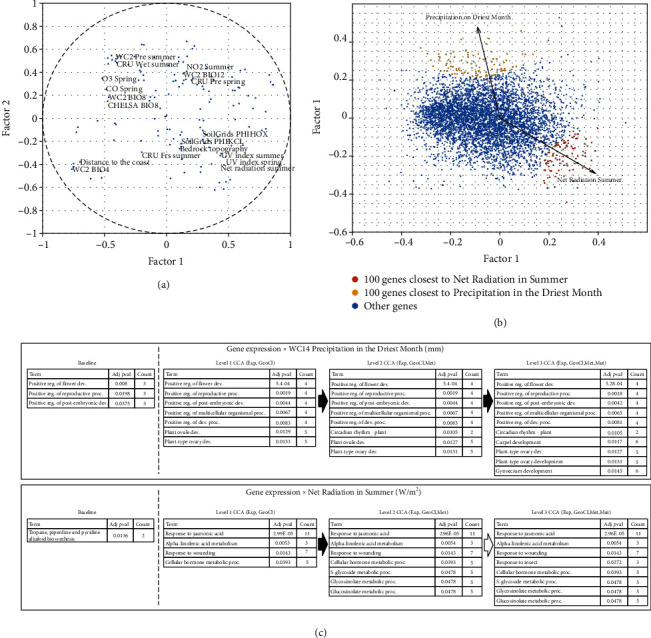
Enrichment analysis of the genes correlated with geoclimatic features. (a) Projection of all the geoclimatic features into the first two canonical factors. A subset of the dots represents labels as examples. (b) Projection of the 5,000 genes in the transcriptome data together with two geoclimatic features: “Net Radiation in Summer” and “Precipitation in the Driest Month”, marked by the arrows. The 100 genes closest to the two geoclimatic features are shown in red and yellow, respectively. (c) The improved enrichment analysis of the 100 closest genes as more levels of HCCA is considered. The baseline results are obtained by directly calculating the correlation between gene expressions and the two geoclimatic features without any projection or coprojection.

The first analysis by only projecting the geoclimatic features to the first two canonical components is shown in Figure 8(a). Consistently, similar geoclimatic features are projected closely. For example, “Ultra Violet (UV) Index in Summer”, “UV Index in Spring”, and “Net Radiation in Summer” appear very close in the projection. Similarly, the abiotic stress features “Ozone (O_3_) level in Spring” and “Carbon Monoxide (CO) level in Spring” also appear close and together with the “BIO8” from the WC2 (WorldClim v2) and CHELSA (Climatologies at High Resolution for the Earths Land Surface Areas). Figure 8(b) shows two geoclimatic features of interest, “Net Radiation in Summer” and “Precipitation in the Driest Month”, projected together with all the genes based on the canonical factor loadings. The 100 genes closest to the “Net Radiation in Summer” are marked red, and the 100 genes closest to “Precipitation in the Driest Month” are marked yellow. These two sets of 100 genes are considered to correlate with the two geoclimatic features in the first 2 factors and further analyzed by clusterProfiler enrichment.

Figure 8(c) at the top shows results of enrichment analysis for the 100 genes closest to “Precipitation in the Driest Month”. The tables show the terms enriched at each level. There are several interesting observations in the enrichment analysis. First, it is evident that CCA is able to enrich more function terms than the baseline which calculates the correlation in the original space without coprojection. For example, it is known that the environment precipitation has an effect on the flower development and reproduction [30]. The enriched term “Circadian rhythm” is known to be related to the flowering and is also affected by environmental changes [31], and the enriched terms “Carpel development”, “Ovary development”, and “Gynoecium development” involve structures that are better developed in the absence of drought [32]. Moreover, some of these terms are only enriched when all the levels of HCCA coprojections are utilized in the analysis.

Figure 8(c) at the bottom shows a similar analysis of “Net Radiation in Summer”. Several terms related to defense response are enriched such as “Response to wounding” and “Response to insect”. It is known that the elevated growth temperature is associated with plant defense responses, which makes the plant more susceptible to pathogens [33, 34]. In addition, the temperature is one of the main environmental factors that affect plant metabolism [35], which explains the terms related to the metabolism process. Finally, the roles of jasmonic acid in temperature stress have been investigated in [36]. HCCA missed the alkaloid term associated with “Net Radiation” which is identified by the baseline method to be associated with this climate feature [37]. Note that this enrichment of alkaloid term only involves 2 genes, AT2G29370 and AT1G31690, with a weak association. Network-based HCCA does capture AT1G31690 and reports a *p* value of 0.08 for the enrichment which is only slightly off the 0.05 cutoff.

**Figure 9:**
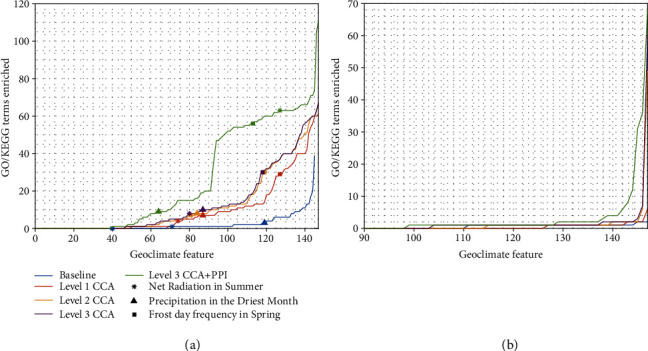
Comparison of the number of enriched terms. The plots show the number of enriched GO and KEGG terms as more datasets and PPI are introduced in the analysis of genes associated with geoclimatic features using (a) gene expressions and (b) DNA methylations. Each curve was sorted by the number of enriched terms. The blue curve shows the baseline which applies correlation between genes and the geoclimatic features in the original sample space. In the plots in (a), three interesting geoclimatic features are labeled in each curve in different shapes for comparison.

It is important to note that in the enrichment analysis of both of the geoclimatic features, the number of the enriched terms and the relevant genes in these enriched terms tend to increase, which significantly improves the accuracy and the confidence of the enrichment analysis.  Figure 9(a) shows the number of enriched terms across all the geoclimatic features, and we can see again that combining more datasets significantly increased the number of terms identified. The results strongly suggest that by incorporating other types of genomic data in the coprojection with gene expressions and geoclimatic features, more useful functional information is introduced into the coprojections. The results confirm the value of integrating multiple datasets for gene-geoclimate association analysis. Additionally, Figure 9(b) shows the same analysis with the DNA methylation profiles. We also observe that the incorporation of multiple genomic datasets in coprojection increases the number of the enriched functional terms while the difference is less obvious.

#### 3.2.2. Incorporating PPI Network Improves Confidence of Enrichment Analysis

We next include the PPI network as a smoothness term in the CCA framework as shown in equation (6) to find a feature representation that considers the interaction between the proteins as well as the correlations between the pairs of the datasets. We still performed HCCA by merging gene expressions and geoclimatic features at the first level and used the condition number to select the datasets at levels 2 and 3 in the hierarchical structure. The selection of parameter *α* follows equation (8).

Figure 9 shows the improvement achieved when utilizing the PPI network in the analysis. The two green curves in Figures 9(a) and 9(b) show that using all the genomic data together with the PPI network significantly increases the number of enriched terms in correlating gene expressions or DNA methylations with the geoclimatic features, respectively. More specifically with gene expressions, the analysis using the PPI network enriched in average 26.2 terms for each geoclimatic feature, against 13.2 terms for running HCCA without the PPI network and 2.9 terms by correlation in the original space.

Figure 9(a) also shows the improvement in the enrichment analysis of two geoclimatic features: “Frost day frequency in Spring” (marked by squares) and “Net Radiation in Summer” (marked by stars) with or without using the PPI network. In the association analysis of “Frost day frequency in Spring”, the number of enriched terms is increased from 30 to 56; and in the analysis of “Net Radiation in Summer”, the number is increased from 8 to 63. The specific terms, as well as the list of genes and statistical confidence levels, can be found in the Supplementary Table 2. In the analysis with the PPI network, the 26 new terms associated with “Frost day frequency in Spring” include “Photosynthesis” as an adaptation to cold stress [38] and “Trichoblast maturation”, which happens in low phosphate condition [39] in need for cold tolerance [40]. Terms related to response to starvation and homeostasis linked to cold response [41, 42] were identified with or without the PPI network. Moreover, more genes are associated to the enriched terms with higher confidence. For example, “Cellular response to starvation” was enriched by 10% of the genes without the PPI network compared with 17% with the PPI network. In the analysis of “Net Radiation in Summer” with the PPI network, compared with the results in Figure 8(c), 55 new enriched terms were identified, including other defense response terms as “Regulation of immune response” and “Defense response to fungus” and other terms linked to temperature stress, as “Salicylic acid mediated signaling pathway” [36].

Overall, the incorporation of the PPI network to guide the coprojections identifies a better set of genes associated with the geoclimatic features of interest, which lead to the identifications of more relevant GO and KEGG terms with higher confidence in the enriched analysis.

## 4. Discussion

In this work, we studied the role of geoclimatic variables in phenotype prediction, in particular the flowering time of *Arabidopsis thaliana* and the interactions between geoclimatic variables and genomic features. To facilitate the study, we proposed HCCA, a hierarchical approach for data integration with canonical correlation analysis. We also adopted several advanced computation techniques including the condition number to measure cross-dataset correlation, graph Laplacian to incorporate network information in coprojection, and hierarchical analysis of canonical factor loadings to detect gene-geoclimate interactions. One advantage of the proposed HCCA framework is the simplicity of model selection where only hyperparameter *c*, the cutoff condition number, is a generic measure in matrix theory. As we show in Supplementary Table 3, we observe consistent results for *c* < 10 in all the CCA-based methods. It is very likely that HCCA is a robust method to generalize to new test data and applications to other dataset integration problems.

It is important to note that our study is different from those on general genotype×environment interactions (G × E) where the environment refers to the growth and development environment of the plant [43]. Thus, our study does not explain or predict the fitness and the adaptation of a genotype in some particular environment. Nevertheless, this study tests the hypothesis that geoclimate features contain useful information for building data-drive predictive models of phenotypes. In this supervised learning setting, the phenotypes of a large number of training genotypes are known; the task is to predict the phenotype of a test genotype. While this setting is not easily applicable in most empirical breeding problems, it provides insights into the impact of geoclimate features from the plant origin on the plant genetic and epigenetic makeups, gene expressions, and their phenotypes, and this association can improve phenotype prediction.

It is a limitation of the current study that the geoclimatic variables do not constitute the growth environment of the individuals, and thus, we cannot affirmatively conclude that the same observation would apply to the environmental information from the grown location. It is likely that origin geoclimate information and growth environment provide complementary information for gene-environment association analysis. One possible future work is to use the phenotypes measured under different environment conditions such as those from the 13 studies in AraPheno as features/outputs to predict each other. Unfortunately, there is no existing well-organized large-scale environment data available, even for *Arabidopsis thaliana* populations, to enable a comprehensive analysis. Nevertheless, HCCA and the other techniques used in this study are all applicable to the analysis, if the data become available in the future.

Another possible limitation is that the gene expression profiles and the methylation profiles in the study were collected at a temperature of 22°C while the target flower time was measured at a temperature of 10°C. Ideally, we would like to have the molecular profiles measured together with the phenotypes at the same temperature. Our results suggest that the transcriptome and methylome collected at 22°C are still very predictive of the FT10. We postulate that the transcriptome collected at 22°C captures the general structure of the underlying biological system and the coordinations among the gene expressions, which is helpful information for predicting other phenotypes including the flower time at different temperatures by supervised learning.

Finally, other than support vector regression, we also tested two more supervised learning methods including deep neural networks and linear regression. We noticed these other methods generated similar or worse prediction results, some of which are shown in Supplementary Figure 2. It should also be possible to integrate other variations of CCA as a building block in our hierarchical approach, such as a structured sparse canonical correlation analysis [44], which incorporates structural information between variables. It is also possible to consider the population structure among the individuals to guide CCA. Typically, the pedigree information of the accessions are better organized as a hierarchical family tree and the tree can be used to derive subgroup for pattern discovery. Since the hierarchical tree built either from pedigree information or genetic information is highly inferable with the mutation data in this study, more detailed analysis of their relation is necessary to motive the integration of a population structure, and in addition, ideally, a different computational technique is needed for incorporating the tree structures such as tree-guided group Lasso [45]. Therefore, it is possible to further optimize these methods but we choose to focus on the data integration aspect in this study and would like to leave the tuning of regressors and other structural regularization models for future work.

## Figures and Tables

**Algorithm 1:**
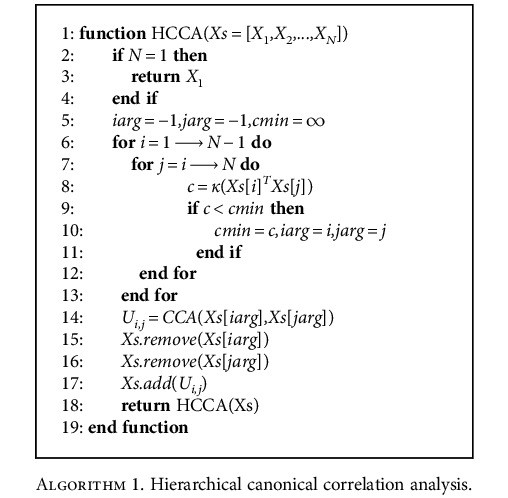
Hierarchical canonical correlation analysis.

**Table 1:** The proportion of predicted cases inside the confidence region.

	Mutation	Expression	Methylation	Climate	HCCA	PCCA	TCCA
5%	0.3154	0.2575	0.2774	0.2834	0.3673	0.2994	0.2794
10%	0.5569	0.5010	0.5170	0.5689	0.6208	0.5948	0.5449
15%	0.7246	0.6866	0.7226	0.7365	0.7984	0.7924	0.7345

## Data Availability

All the source code and data are available at https://github.com/kuanglab/HCCA.

## Supplementary Materials

Supplementary Materials Supplementary Figure 1: integration of pairs of genomic datasets for FT10 prediction—comparison of FT10 prediction by CCA, SNF, and stacking datasets to integrate each pair of the genomic datasets. Supplementary Figure 2: comparison of regression algorithms for FT10 prediction—linear regression, neural network, and support vector regression (SVR) were compared on predicting FT10 values with the projection learned with HCCA integrating a genomic dataset and the geoclimate dataset: (a) gene expression+geoclimate, (b) mutation+geoclimate, and (c) methylation+geoclimate. Supplementary Figure 3: FT16 phenotype prediction results—comparison of the *R*^2^ values for FT16 phenotype prediction with SVR on (a) each individual dataset; (b) integration of a genomic dataset and the geoclimate dataset; (c) all the datasets combined by HCCA, TCCA, PCCA, SNF, and stacking datasets. Supplementary Table 1: geoclimate variables—list of geoclimate variables. Supplementary Table 2: gene enrichment analysis by canonical factor loadings—list of GO terms and KEGG pathways enriched by the genes selected by canonical factor loadings for the geoclimate variables Frost day frequency (Spring) and Net Radiation (Summer). Supplementary Table 3: parameter comparison—FT10 phenotype prediction by HCCA, PCCA, and TCCA when different condition numbers are used for the cross-covariance calculation.
